# Parallel Spatial–Temporal Self-Attention CNN-Based Motor Imagery Classification for BCI

**DOI:** 10.3389/fnins.2020.587520

**Published:** 2020-12-11

**Authors:** Xiuling Liu, Yonglong Shen, Jing Liu, Jianli Yang, Peng Xiong, Feng Lin

**Affiliations:** ^1^College of Electronic Information Engineering, Hebei University, Baoding, China; ^2^Key Laboratory of Digital Medical Engineering of Hebei Province, Hebei University, Baoding, China; ^3^College of Computer and Cyber Security, Hebei Normal University, Shijiazhuang, China; ^4^Beijing Key Laboratory of Mobile Computing and Pervasive Device, Institute of Computing Technology, Chinese Academy of Sciences, Beijing, China; ^5^School of Computer Science and Engineering, Nanyang Technological University, Singapore, Singapore

**Keywords:** motor imagery, EEG, BCI, spatial-temporal self-attention, deep learning

## Abstract

Motor imagery (MI) electroencephalography (EEG) classification is an important part of the brain-computer interface (BCI), allowing people with mobility problems to communicate with the outside world via assistive devices. However, EEG decoding is a challenging task because of its complexity, dynamic nature, and low signal-to-noise ratio. Designing an end-to-end framework that fully extracts the high-level features of EEG signals remains a challenge. In this study, we present a parallel spatial–temporal self-attention-based convolutional neural network for four-class MI EEG signal classification. This study is the first to define a new spatial-temporal representation of raw EEG signals that uses the self-attention mechanism to extract distinguishable spatial–temporal features. Specifically, we use the spatial self-attention module to capture the spatial dependencies between the channels of MI EEG signals. This module updates each channel by aggregating features over all channels with a weighted summation, thus improving the classification accuracy and eliminating the artifacts caused by manual channel selection. Furthermore, the temporal self-attention module encodes the global temporal information into features for each sampling time step, so that the high-level temporal features of the MI EEG signals can be extracted in the time domain. Quantitative analysis shows that our method outperforms state-of-the-art methods for intra-subject and inter-subject classification, demonstrating its robustness and effectiveness. In terms of qualitative analysis, we perform a visual inspection of the new spatial–temporal representation estimated from the learned architecture. Finally, the proposed method is employed to realize control of drones based on EEG signal, verifying its feasibility in real-time applications.

## 1. Introduction

Electroencephalography (EEG) has been widely used in many noninvasive brain–computer interface (BCI) studies because it is simple, safe, and inexpensive (Kübler and Birbaumer, 2008; Lotte et al., 2018). Among the different types of EEG signals, motor imagery (MI) is most commonly used. When people imagine or execute a movement with their hands, both feet, or tongue, the power of the mu (8–12 Hz) and beta (16–26 Hz) rhythms are suppressed or promoted in the sensorimotor region of the contralateral and ipsilateral hemispheres (Pfurtscheller et al., 1997; Pfurtscheller and Da Silva, 1999; Neuper and Pfurtscheller, 2001). Our goal is to classify these MI EEG associated brain activities accurately to allow people with mobility problems to communicate with the outside world via assistive devices.

Numerous studies have examined the classification of MI EEG signals. These studies can be divided into two categories: traditional methods and deep learning-based methods. Among the traditional methods, the common spatial pattern (CSP) algorithm (Müller-Gerking et al., 1999; Ramoser et al., 2000) and its variants are widely used to extract the spatial distribution of features from multi-channel EEG data. The fundamental principle of CSP is to find a set of optimal spatial filters through the diagonalization of a matrix, so as to maximize the difference between the variance values of the two types of signals, and thereby obtain a feature vector with higher discrimination. Filter bank common spatial pattern (FBCSP; Ang et al., 2008) is a variant of CSP that improves the classification accuracy by performing autonomous selection of the discriminative subject frequency range for bandpass filtering of the EEG measurements. Jin et al. (2019) used Pearson's correlation coefficient to manually select the channel that contained the most correlated information, and then employed the regularized common spatial pattern (RCSP) to extract effective features and a support vector machine (SVM) as a classifier. However, the feature selection is heavily reliant on handcrafted features. In addition, because MI EEG signals have limited spatial resolution, a low signal-to-noise ratio (SNR), and highly dynamic characteristics, traditional methods are unable to achieve high decoding accuracy.

Currently, deep learning (DL) exhibits excellent performance in a variety of medical applications (Kumar A. et al., 2016; De et al., 2017; Ma et al., 2017, 2018), and an increasing number of BCI researchers are investigating the use of DL models in MI classification tasks (Schirrmeister et al., 2017). The majority of studies use either feature-based input networks or original signal-based input networks. In the former case, the EEG signals are first transformed from 1D feature vectors into 2D manually specified feature maps by combining spatial, spectral, and temporal information using conventional feature-extraction methods (such as spectrograms and wavelets). The extracted features are then fed into a classification network (Lu et al., 2016; Tabar and Halici, 2016; Zhu et al., 2019). Kumar S. et al. (2016) used CSP to extract features, which were fed into a multilayer perceptron (MLP). Sakhavi et al. (2018) proposed a new feature representation method that combined FBCSP and the Hilbert transform to extract spatial and temporal features. Subsequently, a 5-layer convolutional neural network (CNN) architecture was used for classification. The work (Vaswani et al., 2017) is the first to propose the self-attention mechanism to draw global dependencies of inputs and applies it in machine translation, attention modules are increasingly applied in many flied (Lin et al., 2017; Shen et al., 2018; Fu et al., 2019). However, feature information about the MI signals will be lost when a manually specified feature extraction method is used, which has a negative effect on performance.

Input networks based on the original signal, i.e., the *C* (channel) × *T* (time point) matrices, obtain high-level implicit representations from raw EEG signals without manual feature selection. In such networks, the feature extraction and classification steps are combined in a single end-to-end model with (or without) minimal preprocessing. EEGNet (Lawhern et al., 2018) is a successful network that uses relatively few parameters to achieve good performance on various EEG classification tasks. Azab et al. (2019) proposed a novel weighted transfer learning approach that improves the accuracy of MI classification in BCI systems. Song et al. (2019) improved the classification performance with limited EEG data by combining the representation module, classification module, and reconstruction module into an end-to-end framework. Sakhavi et al. (2018) introduced a new data representation using a spatial–temporal DL model architecture that is designed to learn temporal information from the original input signals. Amin et al. (2019) used a multilayer CNN model that fuses different characteristics of the raw EEG data from the spatial and temporal domains. Zhao et al. (2019) developed a new 3D representation of EEG, a multibranch 3D CNN, and a corresponding classification strategy. Their approach achieved good performance and significantly improved the classification accuracy for different subjects.

Although DL has made remarkable progress in MI classification, it still faces many challenges. First, previous methods mainly select signal channels in motor regions such as C3, Cz, and C4, but MI for different body parts may activate different functional regions of the brain (Ehrsson et al., 2003; Gong et al., 2018). All brain functional areas will have certain effects on the different MI tasks, not only the motor regions. Because the strength of the MI EEG signals varies from person to person, it is impossible to determine exactly which brain regions are most associated with MI (Ma et al., 2020). Second, MI signals are temporally continuous with low SNR and are susceptible to a variety of biological affects (e.g., eye blinks and muscle activity) or environmental artifacts (e.g., noise). Dynamic changes to the EEG signal in the time domain often contain valuable information about the raw MI EEG signals, although these are often neglected by traditional methods, making feature extraction more complicated. The combination of these factors means that previous methods have a limited ability to extract general representations and suffer from low classification accuracy.

To overcome these problems, we propose an end-to-end parallel spatial–temporal self-attention-based CNN for four-class MI EEG signal classification based on the raw MI EEG signals. The proposed method assumes that motor-dependent channels and sampling time steps should be assigned higher weight values than motor-independent channels and sampling time steps during brain activity. The weight values are calculated based on the proposed parallel spatial–temporal self-attention mechanism, which captures high-level distinguishable spatial–temporal features and defines a more accurate compact representation in the space and time domains of the raw MI EEG signal data. Our CNN is capable of modeling high-level, robust, and salient feature representations hidden in the raw EEG signal streams, and can capture complex relationships within data via the stacking of multiple layers of information processing modules in a hierarchical architecture. The major contributions of this study can be summarized as follows:

- In the spatial domain, each channel is recorded from each electrode in various brain areas. We use the spatial self-attention module to capture the potential spatial links between any two channels of the MI EEG signals. The features in a certain channel are updated by aggregating the features over all channels with a weighted summation, where the weights are automatically learned by the feature similarities between the corresponding channels. This module defines a new learned spatial representation of the raw MI EEG data that choose the best channels by automatically assigning higher values to motor-dependent channels and lower values to motor-independent channels. This verifies our assumption that when people think about an action, any channel with similar motor-dependent characteristics can promote mutual improvement, regardless of its spatial location in the brain. As a result, this module improves the classification accuracy and eliminates the artifacts caused by the manual selection of signal channels.
- In the temporal domain, we know that MI EEG signals are continuous with low SNR, which means that there must be a correlation between each time step. Therefore, we use the temporal self-attention module to capture the temporal dependencies between any two sampling time steps, and update each sampling time step using a weighted sum of all sampling time steps. This module defines a new temporal representation of the raw MI EEG data that enhances the temporal representation by encoding the relevant continuous dynamic changes into the global temporal features of each sampling step in the time domain. This is superior to a single-valued representation. In other words, instead of a single sampling value, a new automatically learned temporal representation of the signal is used to extract high-level temporal features from the MI EEG signals in the time domain. Through this module, we assign more weight to the sampling points related to MI and reduce the weight of sampling points that are not related to MI. It is generally believed that there is little useful information in the artifacts, so the temporal self-attention module effectively reduces the interference caused by artifacts.
- The proposed model is evaluated on two challenging datasets to validate its robustness against data variations. The corresponding results demonstrate that our method outperforms several traditional methods (11.09% better on average) and DL-based methods (4.14% better on average) for four-class MI EEG classification by combining spatial and temporal features via the proposed parallel spatial–temporal self-attention architecture. To intuitively verify the rationality of the self-attention mechanism from physiological signals, we plot topographic maps of MI EEG data to illustrate that MI not only activates channels C3, C4, and Cz, but also affects different signal channels. In addition, a BCI application experiment is performed in which we train a model using the data collected in our laboratory and apply it to a drone's online control system based on AirSim (Shah et al., 2018), which is an open source simulator developed by Microsoft.

The remainder of this paper is organized as follows. Section 2 describes the datasets and discusses the details of our method. Experimental results are then presented and use EEG MI to control a drone in section 3. In section 4, we discuss the experimental results from the EEG topographic map. Finally, section 5 presents our conclusions and provides some suggestions for future work.

## 2. Materials and Methods

### 2.1. Overview

In this study, we employed two widely used public EEG MI datasets for evaluation. The main differences between them are the number of channels, trials, subjects, tasks, and sampling rates.

The first dataset is the BCI Competition IV dataset 2a (BCIIV2a) (Tangermann et al., 2012), which recorded a four-class MI task (left hand, right hand, both feet, and tongue) performed by nine subjects across 25 channels (22 EEG and 3 electrooculogram) with a 250 Hz sampling rate. Each channel was preprocessed with a bandpass filter of 0.5–100 Hz. For each subject, two sessions were recorded on different days. Each session comprised six runs separated by short breaks. One run consisted of 48 trials (12 for each of the four possible classes), yielding a total of 288 trials per session. We used one session as the training set, with the other session used to test the classifier and evaluate the performance. Thus, the training set consisted of the 288 trials from the first session and the test set consisted of the 288 trials from the second session. In addition, each trial was extracted using the same time window of [−0.5, 4*s*] on the MI phase of the signals over all 22 EEG channels. Hence, the input signal of our method consists of time series from 22 channels containing 1, 125 sampling points (22 × 1, 125).

The second dataset is the high gamma dataset (HGD) (Schirrmeister et al., 2017), recorded during a four-class MI task across 44 EEG channel signals by 14 healthy subjects performing 4-s trials of certain movements, with 13 runs per subject. The four classes of movements involved the left hand, the right hand, both feet, and rest (no movement). For each subject, the training set consisted of approximately 880 trials (all runs except the last two runs), and the test set consisted of approximately 160 trials (the last two runs). The sampling rate for HGD was 500 Hz. For a fair comparison with BCIIV2a, HGD was resampled to 250 Hz and used the same 4.5-s time window, so that 44 × 1, 125 data points were obtained for each trial.

We performed basic preprocessing of the MI EEG data, such as frequency filtering and normalization. A low-pass filter of 38 Hz and a high-pass filter of 0 Hz were applied to BCIIV2a, and a low-pass filter of 38 Hz and a high-pass filter of 4 Hz to HGD (Schirrmeister et al., 2017). We performed exponential moving standardization to compute the exponential moving means and variances for each channel, and used these to standardize the continuous data.

**Figure 1:**
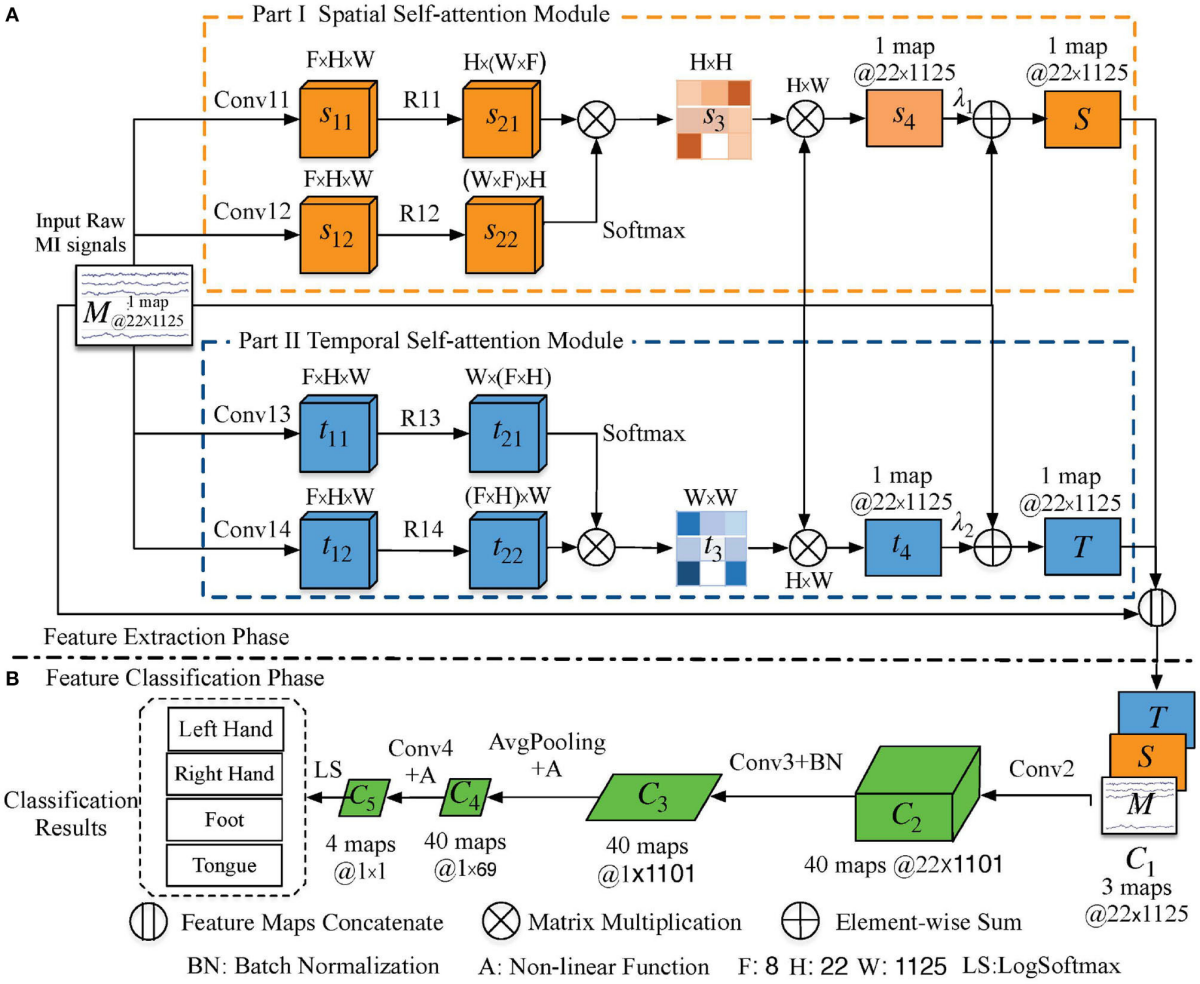
Schematic illustration of the proposed method. The orange, blue, and green cuboids are feature maps in different modules; their corresponding sizes are indicated in the annotation. The convolution and pooling operations are indicated by the arrow lines. **(A)** Parallel spatial–temporal self-attention architecture-based feature extraction phase. The spatial and temporal self-attention modules are denoted by orange and blue rectangles, respectively. **(B)** Feature classification phase. *F* is the number of feature maps, and *H* and *W* are the height and width of the input signal, respectively, which means 22 sampling channels with 1, 125 time steps.

The network architecture, as illustrated in Figure 1, consists of two phases: a feature extraction layer and a feature classification layer. We first describe the feature extraction layer, which contains a parallel spatial–temporal self-attention architecture that extracts distinguishable features in the space and time domains. We then describe how to concatenate the extracted spatial–temporal features for the classification of the MI task and its corresponding training strategy. The code for our model is available in https://github.com/Shenyonglong/Spatial-Temporal-attention-.

### 2.2. Construction of Self-Attention Module

#### 2.2.1. Spatial Self-Attention Module

Traditional approaches usually select EEG channels manually, or assume that each channel plays an equal role. However, the active brain regions for the same MI action are different for different people, which means that the strength of the MI signal varies from subject to subject, as well as for different trials by the same subject. This variation results in low classification accuracy. Therefore, to automatically select the most useful signal channel for extracting discriminant feature representations for subjects and eliminate the artifacts caused by manual selection of signal channels, we propose a spatial self-attention module.

Consider the orange rectangle in Figure 1 and its network parameters in Table 1. Let *M* ∈ ℝ^*H*×*W*^ be the raw data of height (*H*) 22 and width (*W*) 1,125. We first feed these data into two convolution layers (*Conv*11 and *Conv*12) to generate feature maps *s*_11_ and *s*_12_, where *s*_11_ and *s*_12_ belong to ℝ^*F*×*H*×*W*^ and *F* = 8 denotes the number of feature maps. Then, *s*_11_ and *s*_12_ are reshaped (*R*11 and *R*12) to ℝ^*H*×(*F*×*W*)^ and ℝ^(*F*×*W*)×*H*^, respectively, to enable matrix multiplication between them. Finally, a softmax function is applied to obtain the spatial self-attention weight map $s_{3}\in {\mathbb{R}}^{H\times H}$ as:

(1) $$\begin{array}{r} s_{3}^{ij}=\frac{Func\left(s_{21}^{i},s_{22}^{j}\right)}{\sum_{j=1}^{H}Func\left(s_{21}^{i},s_{22}^{j}\right)} \end{array}$$

where *Func* is the similarity function, which uses matrix dot multiplication to calculate the similarity. $s_{3}^{ij}$ denotes the similarity between the *i*th and *j*th channels, and ranges from 0 to 1 (with 0 indicating no similarity and 1 indicating complete similarity).

**Table 1:** Detailed architecture of the proposed spatial self-attention module.

	**Spatial-attention module**						
Input	*M*(22, 1125)	*M*(22, 1125)	*s*_11_(8, 22, 1125)	*s*_12_(8, 22, 1125)	*s*_21_, *s*_22_	*s*_3_, *M*	*s*_4_, *M*, λ_1_
Layer	*Conv*11	*Conv*12	*R*11	*R*12	MMN	MM	ES
Output	*s*_11_(8, 22, 1125)	*s*_12_(8, 22, 1125)	*s*_21_(22, 9000)	*s*_22_(9000, 22)	*s*_3_(22, 22)	*s*_4_(22, 1125)	*S*(22, 1125)
Feature maps	8	8	1	1	1	1	1
Kernel	(1, 1)	(1, 1)	–	–	–	–	–
Stride	(1, 1)	(1, 1)	–	–	–	–	–

*MMN, Matrix multiplication + Normalization + Softmax; MM, matrix multiplication; ES, element-wise sum*.

Matrix multiplication between *s*_3_ and *M*^*H*×*W*^ is performed to obtain the spatial predicted signal $s_{4}\in {\mathbb{R}}^{H\times W}$. Signal *s*_4_ is a spatial predicted signal in which each channel is a weighted sum of other channels from the raw data in the space domain. This task automatically learns similar weights between channels and updates each channel by adaptively aggregating spatial signal data across all channels with the weighted summation. In addition, we perform a residual block by multiplying a learnable parameter λ_1_ by *s*_4_ and perform an element-wise sum operation with the raw signal to obtain the final spatial feature signal (*S* ∈ ℝ^*H*×*W*^) as follows:

(2) $$\begin{array}{r} S=\lambda_{1}\times s_{4}+M \end{array}$$

where λ_1_ is initialized as 0 and is gradually updated to assign more appropriate weights during the training of the whole DL system (Zhang et al., 2019). *S* enhances the representative capability of the inter-subject classification. This means that when people think about an action, any channel with similar characteristics promotes mutual improvement, regardless of its spatial location in the brain.

#### 2.2.2. Temporal Self-Attention Module

MI EEG signals are temporally continuous with a low SNR. Therefore, we constructed a temporal self-attention module (blue rectangle in Figure 1) to generate a temporal predicted signal that is the same size as the raw input data and model the interdependencies between time steps so as to eliminate the artifacts caused by subject and environmental artifacts. The corresponding network parameters are listed in Table 2.

**Table 2:** Detailed architecture of the proposed temporal self-attention module.

	**Temporal-attention module**						
Input	*M*(22, 1125)	*M*(22, 1125)	*t*_11_(8, 22, 1125)	*t*_12_(8, 22, 1125)	*t*_21_, *t*_22_	*M, t*_3_	*t*_4_, *M*, λ_2_
Layer	*Conv*13	*Conv*14	*R*13	*R*14	MMN	MM	ES
Output	*t*_11_(8, 22, 1125)	*t*_12_(8, 22, 1125)	*t*_21_(1125, 176)	*t*_22_(176, 1125)	*t*_3_(1125, 1125)	*t*_4_(22, 1125)	*S*(22, 1125)
Feature maps	8	8	1	1	1	1	1
Kernel	(1,1)	(1,1)	–	–	–	–	–
Stride	(1,1)	(1,1)	–	–	–	–	–

*MMN, Matrix multiplication + Normalization + Softmax; MM, matrix multiplication; ES, element-wise sum*.

The largest difference between this module and the spatial self-attention module is that we reshaped *t*_11_ and *t*_12_ (*R*13 and *R*14) to ℝ^*W*×(*F*×*H*)^ and ℝ^(*F*×*H*)×*W*^ to enable matrix multiplication between them. A softmax function is applied to obtain the temporal self-attention weight map $t_{3}\in {\mathbb{R}}^{W\times W}$ by:

(3) $$\begin{array}{r} t_{3}^{pq}=\frac{Func\left(t_{21}^{p},t_{22}^{q}\right)}{\sum_{q=1}^{W}Func\left(t_{21}^{p},t_{22}^{q}\right)} \end{array}$$

where *Func* is the similarity function, which uses matrix dot multiplication to calculate the similarity. $t_{3}^{pq}$ denotes the similarity between the *p*th and *q*th sampling time steps, and ranges from 0 to 1 (with 0 indicating no similarity and 1 indicating complete similarity). Furthermore, we perform matrix multiplication between the raw signal *M*^*H*×*W*^ and *t*_3_ to obtain the temporal predicted signal $t_{4}\in {\mathbb{R}}^{H\times W}$, which captures the temporal dependencies between any two time steps and updates each time step with a weighted sum of all time steps in the time domain. Finally, a residual block is given by multiplying a learnable parameter λ_2_ by *t*_4_ and performing an element-wise sum operation with the raw signal *M*^*H*×*W*^ to obtain the final temporal feature signal (*T*∈ℝ^*H*×*W*^) by:

(4) $$\begin{array}{r} T=\lambda_{2}\times t_{4}+R \end{array}$$

where *T* encodes the global temporal information into the features of each time step, thus enhancing the representative capability. Therefore, we can extract high-level temporal features of the MI signal in the time domain, thus weakening the artifacts.

### 2.3. Feature Classification

In this section, we describe the concatenation of spatial and temporal feature signals (*S* and *T*) from the raw MI data into the spatial–temporal continuous feature ($C_{1}\in {\mathbb{R}}^{\left(3\times 22\times 1125\right)}$) as:

(5) $$\begin{array}{r} C_{1}=\left\{M,S,T\right\} \end{array}$$

A convolution (*Conv*2) with kernel size 1 × 25 is implemented in the time domain, and then *C*_1_ is fed into the classification network (part b in Figure 1, Table 3). The shape of the output (*C*_2_) is transformed from (3, 22, 1125) to (40, 22, 1101). Furthermore, a convolution (*Conv*3) with kernel size 22 × 1 is applied to the extracted features (*C*_2_) in the space domain. The corresponding shape of output *C*_3_ is (40,1,1101). Third, the average pooling operation (AvgPooling) with kernel size 1 × 75 and stride 1 × 15 is applied over *C*_3_ to generate a coarser feature representation, with the output dimension reduced to (40,1,69). Additionally, the square nonlinear activation is used before the AvgPooling operation and the log nonlinear activation is applied to the output of the AvgPooling operation. All feature maps of *C*_4_ are fed into the final convolution layer (*Conv*4), whose output *C*_5_ has dimensions of (4, 1, 1). Finally, the LogSoftmax function is used to perform multi-classification by converting *C*_5_ to the conditional probability of the four labels.

**Table 3:** Detailed architecture of the proposed temporal self-attention module.

	**Feature classification module**								
Input	*M, S, T*	*C*_1_(3, 22, 1125)	*C*_2_(40, 22, 1101)	*C*_3_(40, 1, 1101)	*C*_3_(40, 1, 1101)	*C*_3_(40, 1, 1101)	*C*_4_(40, 1, 69)	*C*_4_(40, 1, 69)	(4,1,1)
Layer	Concatenate	Conv2	Conv3	Batch normalization	Square	AvgPooling	Log	Conv4	LogSoftmax
Output	*C*_1_(3, 22, 1125)	*C*_2_(40, 22, 1101)	*C*_3_(40, 1, 1101)	*C*_3_(40, 1, 1101)	*C*_3_(40, 1, 1101)	*C*_4_(40, 1, 69)	*C*_4_(40, 1, 69)	(4,1,1)	(4,1,1)
Feature maps	3	40	40	40	40	40	40	40	4
Kernel	–	(1,25)	(22,1)	–	–	(1,75)	–	(1,69)	–
Stride	–	(1,1)	(1,1)	–	–	(1,15)	–	(1,1)	–

### 2.4. Training Strategy

For the four-class MI classification, the NLLoss function in Pytorch was defined as the loss function (Zhu et al., 2018). All parameters in the network were initialized using the Xavier algorithm (Glorot and Bengio, 2010). Adam (Sharma et al., 2017) was employed for the optimization. The learning rate was 0.0001 for the BCIIV2a dataset and 0.001 for HGD. The batch size was 32. Because BCIIV2a and HGD have clearly divided training and test datasets, the training datasets were randomly divided into training (80%) and validation (20%) sets; all test data were selected for the testing stage. This enables us to use the early stopping strategy, developed in the computer vision field, whereby the training set is split into training and validation datasets and the first phase of training stops when the validation accuracy does not improve for a predefined number of epochs. Training then continues on the combined training and validation datasets using the parameter values that led to the best accuracy on the validation dataset. Training ends when the loss function on the validation dataset drops to the same value as that on the training dataset at the end of the first training phase (Schirrmeister et al., 2017). The hyperparameter in the dropout layer and the constant and weight decay rate in the batch normalization layer were set to 0.5, 10^−5^, and 0.1, respectively. All experiments were conducted in Ubuntu 16.04 on a 64-bit system with a Core i9-9900k CPU and 128 GB RAM. Nvidia RTX 2080Ti GPU was utilized for training and testing our model, which was coded using Pytorch and MNE-Python (Gramfort et al., 2014).

### 2.5. Evaluation Metrics

The proposed method was evaluated on two public datasets, BCIIV2a and HGD. The accuracy was used as the evaluation metrics. The accuracy was calculated as:

(6) $$\begin{array}{r} Accuracy=\frac{TP+TN}{TP+TN+FP+FN}\times 100\% \end{array}$$

where TP is the number of true positives, TN is the number of true negatives, FP is the number of false positives, and FN is the number of false negatives.

## 3. Results

To verify the performance and feasibility of our proposed model, we conducted a series of experiments for MI classification on two datasets. The intra-subject classification experiment was intended to verify the performance of the proposed network for an individual subject. The inter-subject transfer experiment was conducted to verify the transfer ability of the proposed method. In this experiment, EEG recordings from other subjects were used to train a model in advance. Next, this model was transferred as the initial weight to further train the individual model.

### 3.1. Quantitative Evaluation of BCIIV2a for Intra-Subject Classification

To confirm the effectiveness and accuracy of the proposed method, we first conducted intra-subject classification using BCIIV2a and compared the accuracy of our method with that given by state-of-the-art DL-based methods [EEGNet (Lawhern et al., 2018), DeepCNN (Schirrmeister et al., 2017), M3DCNN (Zhao et al., 2019), LTICNN (Sakhavi et al., 2018), DMTLCNN (Song et al., 2019), MCCNN (Amin et al., 2019), WTL (Azab et al., 2019)]; traditional FBCSP (Ang et al., 2012) was used as a baseline method to recognize MI EEG data, and an SVM was used as the classifier.

**Figure 2:**
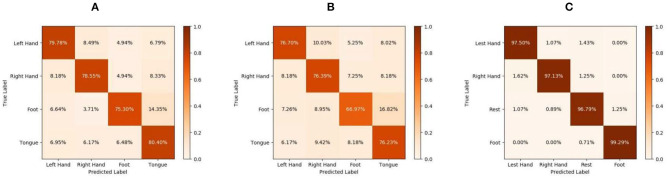
Confusion matrices for the motor imagery (MI) task: **(A)** intra-subject classification of BCIIV2a, **(B)** inter-subject classification of BCIIV2a, and **(C)** intra-subject classification of high gamma dataset (HGD).

Table 4 lists the accuracy of the various methods for each subject and the corresponding average accuracy for the BCIIV2a dataset. Our method clearly outperforms the state-of-the-art DL-based methods, obtaining an average accuracy of 78.51% for the intra-subject classification. Furthermore, confusion matrices for the MI task and the experimental results on the test sets are given in Figure 2A. Although FBCSP provides the best performance for MI signal classification, its average accuracy over all subjects is only 67.42%, which is nearly 11.09% lower than that of the proposed method. Thus, the proposed method yields superior results compared with the traditional machine learning method.

**Table 4:** Accuracy on BCIIV2a for intra-subject classification: comparison between proposed method and other state-of-the-art methods.

	**Accuracy (%)**								
**Subject**	**FBCSP**	**DeepCNN**	**M3DCNN**	**LTICNN**	**DMTLCNN**	**MCCNN**	**WTL**	**EEGNet**	**Proposed**
1	76.00	76.50	77.39	87.50	83.50	**90.21**	90.00	71.88	82.99
2	56.50	50.60	60.14	**65.28**	49.00	63.40	55.00	51.04	56.25
3	81.25	85.00	82.92	90.28	92.70	89.35	93.00	79.17	**93.06**
4	61.00	67.60	72.28	66.67	74.90	71.16	60.00	57.99	**84.03**
5	55.00	72.40	**75.83**	62.50	71.30	62.82	68.00	64.58	68.75
6	42.25	55.10	**68.98**	45.49	63.70	47.66	60.00	51.04	58.34
7	82.75	71.70	76.03	89.58	80.08	**90.86**	73.00	66.32	88.20
8	81.25	74.40	76.85	83.33	80.00	83.72	**98.00**	74.31	88.20
9	70.75	79.20	84.66	79.51	81.70	82.32	83.00	72.57	**86.81**
AVG	67.42	70.28	75.01	74.46	75.21	75.72	75.56	65.43	**78.51**

*Bold font indicates the best scores*.

The DeepCNN model, which is widely used in MI classification algorithms, contains four convolution–pooling block modules. However, because this model is easily overfitted when there are few labeled data for training, the average accuracy of DeepCNN is 70.28%, which is far lower (by 8.23%) than that of our method.

The other DL-based methods compared here are state-of-the-art techniques with excellent representation and accuracy. In this task, their average accuracy values range from 74.46 to 75.72%. LTICNN employs FBCSP as a data preparation method and uses a CNN to extract features. However, because of the need to change the parameters for different subjects, it readily becomes overfitted, and thus achieves worse performance than the proposed method, i.e., 4.05% lower on average. The M3DCNN model combines a new 3D representation of EEG, a multibranch 3D CNN, and a corresponding classification strategy to enhance its resistance to overfitting on different subjects. This model achieves the best results for two subjects (5 and 6) and demonstrates better performance than LTICNN. The greatest contribution of M3DCNN is to demonstrate that a deeper and more complex representation of EEG can help improve the performance. However, its accuracy of 75.01% is 3.50% lower than that of our method. DMTLCNN and WTL employ transfer learning techniques to yield a remarkable increase in classification accuracy, reaching 75.21 and 75.56%, respectively. Compared with this performance, our model is 3.30 and 2.95% more accurate, respectively. The MCCNN model first fuses different CNN modules to prove that the spatial and temporal features can improve the classification over handcrafted features. Previously, MCCNN has achieved the highest accuracy, reaching 75.72%. However, this model focuses on the spatially invariant features of MI EEG signals, and does not consider the interrelationship between the temporal features and spatial features in depth. This interrelationship is the focus of this study. Compared with MCCNN, our average accuracy is 2.79% higher. In addition to the average accuracy, we achieved the best results for three of the nine subjects (3, 4, and 9).

The above results show that the proposed method outperforms all traditional (11.09% better on average) and DL-based methods (4.14% better on average) for four-class MI EEG classification by combining spatial and temporal features via the proposed parallel spatial–temporal self-attention architecture.

For the evaluations using 10-fold cross-validation, we combined the training and testing set of BCIIV2a, and then randomly divided into 10 equal parts. In each run, nine subsets were used as training set and 1 subset was used as the testing set. That means there are 518 trials for training, 58 trials for testing. The final accuracy was obtained by averaging the best values of the 10-fold. Compared with the 288 trials we used for training and 288 trials for testing before, the 10-fold cross-validation significantly increases the amount of the training set, so it can achieve better accuracy (90.15% on average) in Table 5.

**Table 5:** Intra-subject classification of 10-fold cross-validation results on the BCIIV2a.

	**Accuracy (%)**									
**Subjects**	**1**	**2**	**3**	**4**	**5**	**6**	**7**	**8**	**9**	**AVG**
Accuracy	93.93	75.33	97.74	86.46	89.94	78.32	99.14	94.43	96.02	90.15

### 3.2. Quantitative Evaluation of BCIIV2a for Inter-Subject Transfer Learning Classification

One of the main contributions of the proposed method is to improve the accuracy of inter-subject classification through the parallel spatial–temporal self-attention architecture. This is the first time the attention mechanism has been used to study the relationship between channels. Here, by using transfer learning techniques, we utilize the other subjects' EEG data to train a model on the BCIIV2a dataset, and then apply this model as the initial weight of the network and load data from a new subject for further training. In this way, we can consider the trained model to integrate the information of other subjects, thus making it more robust.

Table 6 presents the corresponding classification results for each subject. Because of the large differences between subjects, the results in Table 6 are not better than the intra-subject results (listed in Table 4). Figure 2B shows the confusion matrices for the BCIIV2a dataset inter-subject classification results. Compared with other state-of-the-art DL-based methods (MCCNN, DeepCNN, and DMTLCNN, which provided the inter-subject comparison results), the proposed method obtains the best average accuracy of 74.07%, with particularly good results for five of the nine subjects (1, 3, 6, 7, and 8). The results show that our method not only weakens the artifacts caused by manually selecting a signal channel, but also automatically provides a more robust and generic feature representation with higher classification accuracy of MI EEG signals.

**Table 6:** Results of inter-subject transfer learning classification using the BCIIV2a dataset.

	**Accuracy (%)**									
**Subjects**	**1**	**2**	**3**	**4**	**5**	**6**	**7**	**8**	**9**	**AVG**
MCCNN	62.07	42.44	63.12	52.09	49.96	37.16	62.54	59.32	69.43	55.34
DeepCNN	78.80	**51.80**	86.80	**71.60**	**68.70**	64.60	82.30	80.90	75.40	73.40
DMTLCNN	80.30	50.30	85.50	70.60	66.20	60.60	83.00	82.80	**78.4**	73.10
Proposed	**82.99**	45.84	**94.10**	67.37	54.84	**75.72**	**85.07**	**87.85**	73.27	**74.07**

*Bold font indicates the best scores*.

### 3.3. Quantitative Evaluation of HGD for Intra-Subject Classification

To further verify the adaptability of the proposed method, we conducted intra-subject classification evaluations on another challenging dataset (HGD). In Schirrmeister et al. (2017), we set the low cut-off frequency of HGD to 4 Hz. Because some state-of-the-art methods only report average accuracy values for HGD, we only list the average accuracy in Table 7.

**Table 7:** Intra-subject classification results using high gamma dataset (HGD).

**Accuracy (%)**					
**FBCSP**	**DeepCNN**	**MCCNN**	**CPMixedNet**	**MSFBCNN**	**Proposed**
90.90	91.40	95.40	93.70	94.40	**97.68**

*Bold font indicates the best scores*.

Recall that the tasks used to construct HGD and BCIIV2a are different. The tasks performed for BCIIV2a involve the left hand, right hand, both feet, and tongue, which are different from the four categories of HGD. Additionally, HGD contains much more data than BCIIV2a. As we know, the amount of data is an important factor affecting the performance of DL. Thus, these data allow the proposed method to attain evaluation results that are even more encouraging, with a significant improvement over the other methods. The proposed method reaches 97.68% accuracy, while the second-best method (MSFBCNN; Wu et al., 2019) can attain 94.40% accuracy. The final classification accuracy of MSFBCNN is lower than that of our method because it focuses on multi-scale convolution in the temporal domain, and ignores the spatial relationship between channels. CPMixedNet (Li et al., 2019) also analyzes the time domain, using regular and dilated convolution to extract the temporal EEG information. The classification accuracy is 93.70% after amplitude-perturbation data augmentation. However, there is no analysis of the spatial distribution of EEG information, so the accuracy is 3.98% lower than that of our method. These experimental results demonstrate the capability of the proposed network with data augmentation for MI EEG signal classification. In Figure 2C, we show the accuracy for each class of HGD in the form of confusion matrices.

### 3.4. Control of the Drone Based on EEG Signal

We further tested and validated the real-time capability of the proposed model through the online decoding of MI movements from streamed EEG signals for virtual drone control. We used a Greal 32-channel EEG amplifier developed by Neuroscan to collect the MI EEG data. First, the subjects were asked to imagine writing a Chinese character with their left or right hand according to the paradigm of Qiu et al. (2017). At the beginning of a trial (t = 0 s), a fixation cross appeared on the black screen. At t = 2 s, the fixation cross was replaced by a picture of the forearm and a Chinese character. Each subject had 6 s to perform the MI task, in which hand movements followed the strokes of the Chinese character on the screen. Subjects then had a short break of 2 s. Second, a band-pass filter of 4–38 Hz was applied to the EEG signals. Third, the preprocessed data were sent to the trained network for classification. Finally, the AirSim-based virtual unmanned aerial vehicle (UAV or drone) was directed to move either left or right according to the decoded movement from the EEG signals.

**Figure 3:**
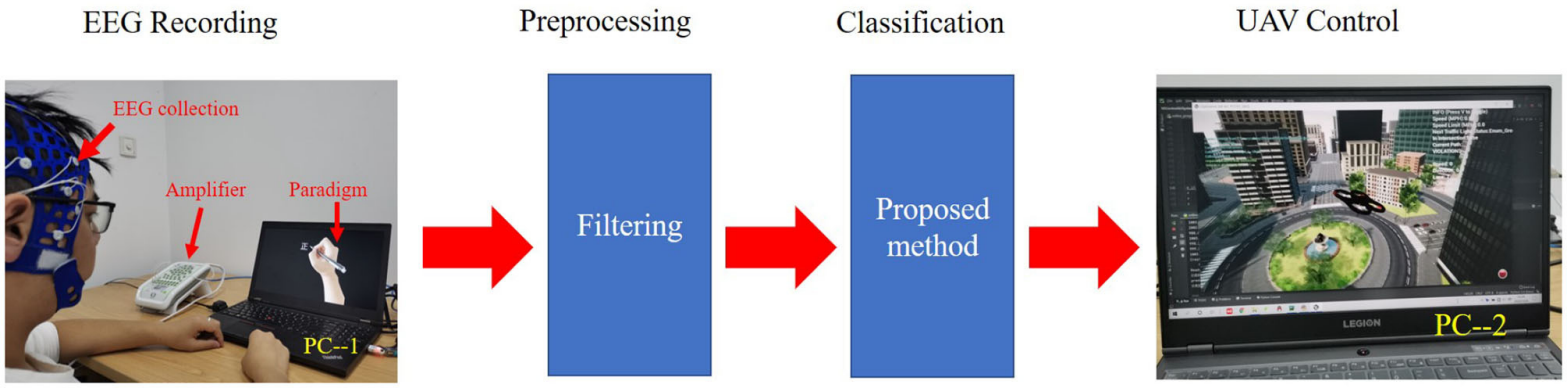
Live setup for real-time electroencephalography (EEG) signal decoding and unmanned aerial vehicle (UAV) control. PC–1 is a computer used to display the paradigm. The subjects only looked at PC–1 during the experiment. PC–2 is a computer used to classify and control the UAV. The classification model was trained in advance.

Figure 3 shows the whole process of how we used the data collected in the laboratory to control the flight of the virtual UAV. When the subjects imagined writing with their left hand, the virtual UAV would fly to the left. Similarly, when the subjects imagined writing with their right hand, the UAV would fly to the right. A video of a successful live demo is available in the Supplementary Materials.

## 4. Discussion

This study has investigated the design and training of an end-to-end neural network using raw EEG signals. This is the first time that a new spatial–temporal representation of raw EEG signals has been defined using the self-attention mechanism for extracting the distinguishable spatial–temporal features. Through intra- and inter-subject transfer learning experiments on the BCIIV2a dataset and HGD, we demonstrated that the proposed method outperforms several state-of-the-art methods in terms of the classification accuracy. At the same time, we visualized topographic maps of MI EEG data to explain the rationality of our temporal and spatial attention mechanism from the perspective of physiological EEG characteristics. Finally, as reported later in this section, we applied this method to control the flight of a UAV using MI EEG data.

**Figure 4:**
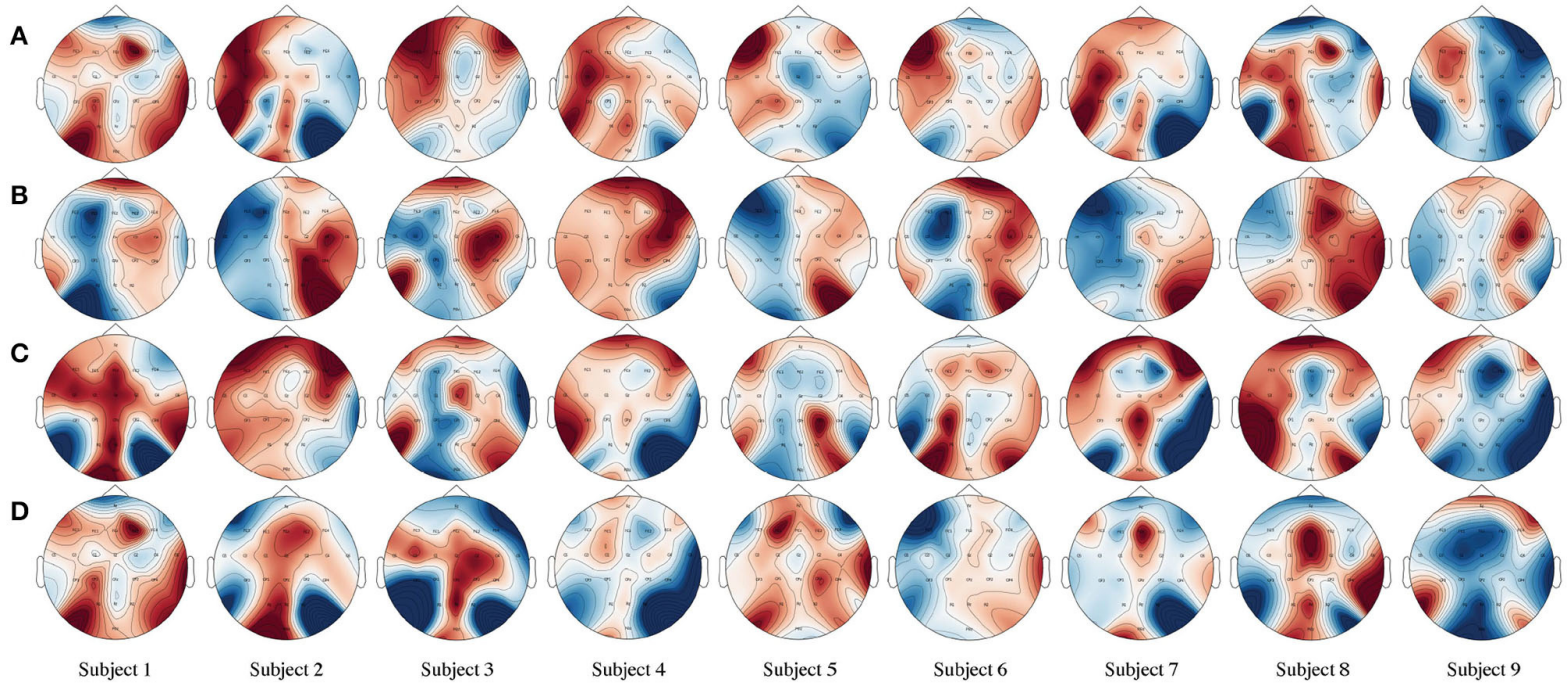
Brain active correlation map corresponding to our classification results for each subject using four-class motor imagery (MI) unmanned aerial vehicle (EEG) signals from the BCIIV2a dataset. Twenty-two Ag/AgCl electrodes were used to record the EEG. **(A–D)** The MI of the left hand, right hand, both feet, and tongue for each line in the picture. The electrode *C*_*z*_ is located on the center of the head. *C*_3_ and *C*_4_ are located on the left and right sides, respectively. These electrodes are positioned directly over the motor cortex areas. Red indicates a positive correlation, i.e., increasing amplitude values (ERS), whereas blue indicates a negative correlation, i.e., decreasing values (ERD).

In Figure 4, we present a brain active correlation map corresponding to our classification results for each subject using four-class MI EEG signals from the BCIIV2a dataset. As we know, when people imagine or execute movements of their left or right hand, both feet, and tongue, the power of the mu (8–12 Hz) and beta (16–26 Hz) rhythms can decrease or increase in the sensorimotor region of the contralateral and ipsilateral hemispheres. The red color indicates a positive correlation, i.e., event-related synchronization (ERS), with a deeper color denoting a stronger positive correlation. In contrast, the blue color indicates a negative correlation, i.e., event-related desynchronization (ERD), with a deeper color denoting a stronger negative correlation. For example, the first row in Figure 4 shows the brain activation pattern for MI data corresponding to left-hand motion. Our classification results are for the MI EEG signals of left-hand motion, and the corresponding brain active correlation map shows the ERS and ERD in the left and right hemispheres.

In addition, the evaluation results shown in Figure 4 prove the validity of our assumption that when people think about an action, any channel with similar motor-dependent characteristics can promote mutual improvement, regardless of its spatial location in the brain. Taking the left-hand MI as an example, the traditional method often manually chooses *C*_3_, *C*_4_, and *C*_*z*_ as inputs. However, as shown by the brain active correlation map for the left-hand MI of subject 1 in Figure 4, in neurophysiological terms, channels FC3, FCz, FC2, C5, Cz, CP1, and CP4 all exhibit the same ERS trend as channel *C*_3_, which is located in the motor cortex area. Similarly, channels Fz, FC4, C2, CPz, and Pz exhibit the same ERD trend as channel *C*_4_. This proves that our initial hypothesis is correct.

Therefore, different from the traditional method, we use the spatial self-attention module to capture the potential spatial links between any two channels of the MI EEG signals. The features within a certain channel are updated by aggregating the features across all channels with a weighted summation, where the weights are automatically learned according to the feature similarities between the corresponding channels. This module defines a new learned spatial representation of the raw MI EEG data that chooses the best channels by automatically assigning higher values to motor-dependent channels and lower values to motor-independent channels. The evaluation results show that our method effectively improves the accuracy of classification.

## 5. Conclusion

This paper has described a parallel spatial–temporal self-attention CNN-based architecture for four-class MI EEG classification. The self-attention mechanism is first introduced for capturing robust and generic feature dependencies in the spatial and temporal dimensions. As a result, we can extract distinguishable spatial–temporal features of MI signals. The experimental results on two public datasets show that the proposed model outperforms several state-of-the-art methods. Furthermore, successful real-time control of a virtual UAV was achieved using the trained model. In the future, we plan to explore the multi-task analysis of MI EEG signals.

## Data Availability Statement

The original contributions presented in the study are included in the article/Supplementary Materials, further inquiries can be directed to the corresponding author/s.

## Author Contributions

XL and YS processed and analyzed the data and wrote the manuscript. JL developed the parallel spatial-temporal self-attention CNN method. JY helped in data analysis. PX helped in manuscript editing. JL and FL supervised development of work, helped in manuscript edit, and evaluation.

## Conflict of Interest

The authors declare that the research was conducted in the absence of any commercial or financial relationships that could be construed as a potential conflict of interest.

## Supplementary Material

The BCIIV2a dataset can be found at: http://www.bbci.de/competition/iv/, and HGD can be found at: https://gin.g-node.org/robintibor/high-gamma-dataset/.
