# Supplementary material for: Parallel Spatial–Temporal Self-Attention CNN-Based Motor Imagery Classification for BCI
Source: Front Neurosci. 2020 Dec 11;14:587520. doi: 10.3389/fnins.2020.587520 (PMC7759669; doi:10.3389/fnins.2020.587520)
Supplement: Supplementary file 2 [file Data_Sheet_1.pdf]

Supplementary Material

1 SUPPLEMENTARY RESULT OF HIGH GAMMA DATASET FOR INTER-SUBJECT TRANSFER LEARNING CLASSIFICATION

In the high gamma dataset (HGD), we also conducted an inter-subject transfer learning experiment to verify the effectiveness of our method. Same as article description, we set the low cut frequency of HGD as 4Hz. Since some state-of-the art methods currently do not provide their inter-subject transfer learning experimental results in the HGD, we do not compare the accuracy of their methods in Table S1, but show the accuracy of our experiment as the supplementary material. Similarly, Figure 1 shows the confusion matrix of HGD inter-subject transfer learning experiment, in which the specific classification accuracy of each class is shown. The confusion matrix in Figure S1 represents the classification accuracy for each class.

Table S1. Inter-subject classification results on the HGD.

	Accuracy (%)						
subjects	1	2	3	4	5	6	7
Acc	99.37	95.00	99.37	100	100	98.75	96.86
subjects	8	9	10	11	12	13	14
Acc	98.75	99.37	96.25	98.75	98.75	97.48	78.12
AVG	96.92						

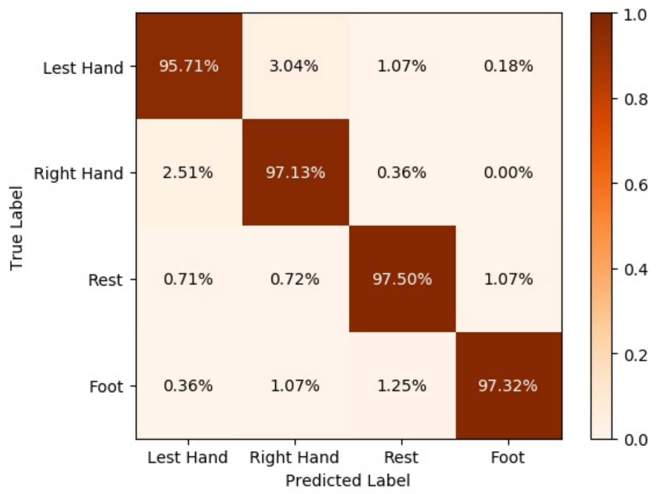

Figure S1. The confusion matrix of motor imagery task on HGD for inter-subject transfer learning classifiction.
